# Antitrypanosomal Activity and Molecular Docking Studies of Lobetyolin From *Lobelia rhynchopetalum* Hemsl. Root Extract Against *Trypanosoma congolense* Field Isolates

**DOI:** 10.1155/bmri/2214310

**Published:** 2026-02-04

**Authors:** Selamawit Yimer, Eyael Tewelde, Daniel Bisrat, Solomon Tadesse, Mariamawit Y. Yeshak

**Affiliations:** ^1^ Department of Pharmacognosy, School of Pharmacy, College of Health Sciences, Bahir Dar University, Bahir Dar, Ethiopia, bdu.edu.et; ^2^ Department of Pharmaceutical Chemistry and Pharmacognosy, School of Pharmacy, College of Health Sciences, Addis Ababa University, Addis Ababa, Ethiopia, aau.edu.et; ^3^ Alfred E. Mann School of Pharmacy and Pharmaceutical Sciences, University of Southern California, Los Angeles, CA 90033, USA, usc.edu; ^4^ Department of Biomedical and Pharmaceutical Sciences, College of Pharmacy, Idaho State University, Pocatello, Idaho, USA, isu.edu

**Keywords:** antitrypanosomal, *Lobelia rhynchopetalum*, lobetyolin, polyacetylene, *Trypanosoma congolense*, trypanothione reductase (TR)

## Abstract

Currently available drugs for trypanosomiasis are few, and their use is limited by toxicity and growing resistance. This highlights the need for safer and more effective alternatives. In this study, the in vitro and in vivo antitrypanosomal activities of an 80% methanol root extract of *Lobelia rhynchopetalum* and its major constituent were evaluated against *Trypanosoma congolense* field isolates. Phytochemical separation of the extract yielded lobetyolin (a polyacetylene compound), confirmed through spectroscopic analysis. At 4 mg/mL, both the crude extract and lobetyolin inhibited parasite motility, with lobetyolin acting slightly faster (25 min) than the extract (30 min). The blood incubation assay demonstrated dose‐dependent protection, with 4 mg/mL of DA, the lobetyolin‐rich extract, or pure lobetyolin preventing infection, while lower doses only delayed parasitemia. In vivo testing showed that lobetyolin at 100 mg/kg exhibited stronger activity than the crude extract at 400 mg/kg. Molecular docking demonstrated that lobetyolin binds strongly to the homology‐modeled trypanothione reductase (TR) enzyme of *T. congolense*, achieving a Glide score of −8.002 kcal/mol, which is close to that of the native ligand (−8.307 kcal/mol). This suggests that lobetyolin may inhibit TR, thereby disrupting the parasite′s redox balance essential for survival. Overall, these findings provide the first evidence supporting the antitrypanosomal activity of *L. rhynchopetalum* root extract and lobetyolin, providing scientific support to the plant′s traditional use. Further studies are needed to fully validate its therapeutic potential.

## 1. Introduction

Neglected tropical diseases (NTDs) impose a significant health, economic, social, and psychological burden on impoverished populations in Africa, Asia, and Latin America [1]. Human, domesticated animal, and wildlife trypanosomiasis present a major global health threat [2]. The limited availability of medications for treating human and animal trypanosomiasis, the increasing prevalence of drug resistance, and the toxicity of existing trypanocidal drugs further complicate disease management [3]. Currently, only six drugs are approved for treating African animal trypanosomiasis, but their low therapeutic indices restrict their use, especially when low‐level resistance emerges [4]. Therefore, there is an urgent need for new drugs that are affordable, effective across all stages of the disease, and capable of eliminating all strains and species of trypanosomes to improve the management of sleeping sickness.

*Lobelia* is a genus of flowering plants belonging to the family Campanulaceae and contains about 415 species with a cosmopolitan distribution [5]. There are 18 species of *Lobelia* in Ethiopia, of which seven species are endemic: *Lobelia rhynchopetalum*, *Lobelia achrochilus*, *Lobelia schimperi*, *Lobelia exilis*, *Lobelia erlangeriana*, *Lobelia tripartite*, and *Lobelia scebelii* [6]. *Lobelia* species are mostly annuals and herbaceous perennials, which differ from small soft herbs to woody plants with rosette leaves (giant lobelias) [7]. The chemistry and biological activities of the genus were systematically reviewed in 2019 [8].

*L. rhynchopetalum* is endemic to the Afro‐Alpine zones of the Semien and Bale Mountains National Parks of Ethiopia at altitudes of 3000–4350 m [8]. In Ethiopian traditional medicine, *L. rhynchopetalum* is commonly used to treat protozoal diseases [9, 10]. Despite these claims, the plant has not been the subject of any investigations. This study is aimed at evaluating the in vitro and in vivo antitrypanosomal activity of *L. rhynchopetalum* root extract and its major constituent. Additionally, the isolated compound was further analyzed using molecular docking techniques to explore its potential mode of action.

## 2. Materials and Methods

### 2.1. Chemicals and Instruments

The chemicals, reagents, and drugs used in this experiment included chloroform (Finkem Laboratory Reagent, India), methanol (Sigma‐Aldrich, United States), and silica gel–based materials for chromatography: analytical TLC (Silica gel 60 F254, 0.2 mm), column chromatography (CC) (Silica gel 60, 70–240 mesh, Merck, Germany), and preparative TLC (Silica gel HF 254, 0.50 mm, LOBACHEMIE, India). Other reagents included dimethyl sulfoxide (DMSO), phosphate‐buffered saline (PBS) (Gibco, United States), microscopic oil, Giemsa stain, 40% glucose (Pharma Cure, Ethiopia), and sterile water (AAU Pharmaceutics Lab). The drug used was diminazene diaceturate (DA) BP Vet [4, 4‐(diazoamino) dibenzamidine diaceturate] with 1.31 g phenazone BP (Ashish, India). All chemicals and reagents were used as received. The instruments used included a rotary evaporator (Heidolph Instruments GmbH & Co., Germany), NMR spectrometer (Bruker Advance 400 FT‐NMR spectrometer [400.13 MHz for ^1^H and 100.6 MHz for ^13^C]), mass spectrometry (Agilent 1100 series system with ESI‐API ionization [capillary voltage: 4000 V, fragmentor: 160 V, drying gas: 350°C, gas flow: 10 l/min, nebulizer pressure: 50 PSIG]). The purity of the isolated compound was confirmed by an Agilent LC/MS system with a diode array detector (DAD).

### 2.2. Plant Collection and Authentication

The roots of *L. rhynchopetalum* were collected from the Semien Mountains in the North Gondar Zone of Ethiopia in April 2021. A voucher specimen (LR‐01) was deposited at the National Herbarium, Department of Biology, College of Natural and Computational Sciences, Addis Ababa University (AAU), where the plant′s leaves, stem, and flowers were authenticated.

### 2.3. Test Organisms and Experimental Animals

*Trypanosoma congolense* was obtained from naturally infected cattle in Abulo Kebele, Arba Minch, Ethiopia. Blood samples were collected from the ear vein using heparinized capillary tubes, centrifuged at 12,000 rpm for 10 min, and examined under a 40× objective microscope for parasite motility. Giemsa staining confirmed the presence of *T. congolense* under a 100× oil immersion microscope. Infected blood was also collected from the jugular vein using an EDTA syringe, and 0.2 mL was injected intraperitoneally (i.p.) into five healthy mice. The parasites were maintained through serial blood passage between infected and noninfected mice. White Swiss albino mice (22–30 g, 5–6 weeks old) from the Department of Pharmacology, School of Pharmacy, AAU, were used. The mice were housed in stainless steel cages under a 12‐h light/dark cycle with ad libitum access to food and water. All procedures followed internationally accepted guidelines for laboratory animal care [11, 12], with ethical clearance obtained from the Ethical Review Committee of the School of Pharmacy, College of Health Sciences, AAU (ERB/SOP/417/14/2022).

### 2.4. Preparation of Plant Extract

Fresh *L. rhynchopetalum* roots were washed, shade‐dried, and crushed. The powdered material (375 g) was macerated in 80% methanol at room temperature for 72 h with continuous agitation. After filtration through sterile gauze and Whatman No. 1 filter paper, the process was repeated twice. The filtrates were combined and concentrated using a rotary evaporator at 40°C, followed by freeze‐drying, yielding a reddish–brown solid substance (18.75 g). The dried extract (80% methanol extract of *L. rhynchopetalum* [LRE]) was stored in an amber bottle at 4°C until use.

### 2.5. Isolation of a Compound by CC

The compound was isolated from the LRE using CC. A silica gel slurry was prepared by mixing silica gel (particle size: 0.2–0.5 mm) with CHCl_3_, packed into the column, and conditioned overnight. The sample, dissolved in methanol, was adsorbed onto silica gel, dried, and loaded onto the column. Elution started with 100% CHCl_3_, followed by a gradual increase in MeOH. Fractions eluted with CHCl_3_/MeOH (9:1) and (8:2) displayed a single spot on TLC. These fractions were combined and evaporated, yielding a yellow solid (LR‐1), which was stored in an amber vial at 4°C until use.

LR‐1: Yellow solid substance; *R*_*f*_ = 0.58 on a silica gel‐TLC using a mixture of CHCl_3_/MeOH (4:1) as a solvent system; HRESI‐TOF‐MS (+ve mode): *m*/*z* = 397.1942 [M + H]^+^ (calcd = 397.1862) and *m*/*z* = 793.3812 [2 M + H]^+^ (calcd = 793.3647), indicating a molecular formula of C_20_H_28_O_8_; ^1^H‐NMR (ppm, CD_3_COCD_3_): *δ* 1.64 (*q*, *J* = 6.9 Hz, 2H, H‐13), *δ* 1.83 (*dd*, *J* = 6.9, 1.9 Hz, 3H, H‐1), *δ* 2.19 (*dd*, *J* = 7.5 Hz, 2H, H‐12), *δ* 3.27 (*m*, 1H, H‐2 ^′^), *δ* 3.29 (*m*, 1H, H‐5 ^′^), *δ* 3.34 (*dt*, *J* = 23.1, 8.6 Hz, 1H, H‐4 ^′^), *δ* 3.41 (*m*, 1H, H‐3 ^′^), *δ* 3.59 (*m*, 2H, H‐14), *δ* 4.28 (*m*, *J* = 7.9, 6.0 Hz, 1H, H‐9), *δ* 3.86 (*dd*, *J* = 12.3, 2.8 Hz, 1H, H‐6_b_^′^), *δ* 4.41 (*d*, *J* = 7.8 Hz, 1H, H‐1 ^′^), *δ* 4.47 (*d*, *J* = 6.1 Hz, 1H, H‐8), *δ* 5.50 (*dd*, *J* = 15.6, 8.0, 1.5 Hz, 1H, H‐10), *δ* 5.65 (*dd*, *J* = 15.5, 1.8 Hz, 1H, H‐3), *δ* 5.95 (*dt*, *J* = 14.4, 6.9 Hz, 1H, H‐11), *δ* 6.38 (*dq*, *J* = 16.0, 6.9 Hz, 1H, H‐2); ^13^C‐NMR (ppm, CD_3_COCD_3_): *δ* 18.06 (C‐1), *δ* 28.74 (C‐12), *δ* 32.06 (C‐13), *δ* 60.92 (C‐14), *δ* 61.80 (C‐6 ^′^), *δ* 65.47 (C‐8), *δ* 69.60 (C‐7), *δ* 70.56 (C‐4 ^′^), *δ* 71.82 (C‐6), *δ* 73.68 (C‐2 ^′^), *δ* 76.95 (C‐5), *δ* 76.95 (C‐5 ^′^), *δ* 76.95 (C‐3 ^′^), *δ* 80.70 (C‐9), *δ* 81.25 (C‐4), *δ* 99.82 (C‐1 ^′^), *δ* 109.30 (C‐3), *δ* 125.26 (C‐10), *δ* 137.29 (C‐11), *δ* 144.39 (C‐2); ^1^H and ^13^C‐NMR spectral data match with those reported for lobetyolin [13].

### 2.6. Acute Oral Toxicity Test

An acute oral toxicity test for the LRE and the compound (LR‐1) from *L. rhynchopetalum* roots was conducted following OECD guidelines [14]. Twelve female albino mice (6–8 weeks old, 24–28 g) were used. The mice fasted for 4 h before and 2 h after administration. Initially, a single mouse received 2000 mg/kg of LRE or LR‐1 via oral gavage. Since no toxicity signs or mortality were observed within 24 h, four additional mice per sample received the same dose. The mice were monitored continuously for 4 h at 30‐min intervals and then daily for 14 days for toxicity signs, appetite loss, and mortality. No adverse effects were observed.

### 2.7. In Vitro Antitrypanosomal Activity

Antitrypanosomiasis activity was assessed using a 200 *μ*L of blood containing approximately 20–25 parasites per field that was combined with 50 *μ*L of test substances at concentrations of 20, 10, 2, and 0.5 mg/mL. This resulted in final test concentrations of 4, 2, 0.4, and 0.1 mg/mL, respectively, in 96‐well plates. The negative control consisted of parasites suspended in 50 *μ*L of 1% DMSO, while the positive control was treated with the same doses of DA [15, 16]. The 96‐well plates were incubated at 37°C for 5 min. Then, 2 *μ*L of the test mixtures was placed on separate microscope slides, covered with a 22 × 22 mm coverslip, and examined every 5 min for 1 h to monitor parasite motility [17]. The reduction or inhibition of parasite motility in the test samples relative to the negative control was used to determine the trypanocide activity [18–20]. This experiment was conducted in triplicate, with each test concentration repeated twice [21, 22].

### 2.8. Blood Incubation Infectivity Assay

The in vivo infectivity test was conducted to determine whether any *Trypanosoma* parasites remained viable after the in vitro blood incubation assay. From each test concentration (4, 2, 0.4, and 0.1 mg/mL), 0.2 mL of the remaining incubation mixture was injected i.p. into healthy mice (five per group). The animals were then observed for 30 days for signs of infection. Blood smears were checked regularly, and changes such as weight loss, reduced activity, or ruffled fur were recorded. The presence of parasites or illness indicated surviving infective parasites, while no infection suggested complete loss of viability in accordance with previously described methods [16, 23].

### 2.9. In Vivo Antitrypanosomal Activity

A total of 45 mice were used in this experiment. Forty mice were injected i.p. with 0.2 mL of *T. congolense*‐infected blood, diluted in PBS, containing approximately 10^5^ trypanosome cells. The infected blood was obtained from donor mice via cardiac puncture [22]. The mice were monitored for 2 weeks to allow parasitemia to develop, reaching an average parasitemia level of approximately 7.20 (log scale) or ~107.20/mL [18]. After this period, the mice were randomly divided into eight groups, each consisting of five mice. Treatment began on Day 14 postinfection (designated as Day 0 of treatment). The groups were assigned as follows: Group 1 received the vehicle (1% DMSO, 10 mL/kg/day) and served as the negative control. Group 2 received a single dose of diminazene aceturate (DA) (28 mg/kg) as the positive control. Groups 3–5 received 100, 200, and 400 mg/kg/day of LRE, respectively. Groups 6–8 received 25, 50, and 100 mg/kg/day of LR‐1, respectively [17].

### 2.10. Determination of Parasitemia

Parasitemia was monitored every other day using a thin blood smear prepared from a drop of blood collected from the mouse tail. The smear was examined under a microscope at 40× total magnification. Parasitemia levels were determined using the rapid matching technique [24]. To ensure accuracy, wet blood smears were prepared in triplicate for each mouse, and the mean slide count was recorded for each sample.

### 2.11. Determination of Packed Cell Volume (PCV), Body Weight, and Rectal Temperature

PCV was measured using a microhematocrit centrifuge, while body weight and rectal temperature were recorded at four time points: before infection (preinfection), before treatment initiation (Day 0), at the end of treatment (Day 7), and at the end of the experiment (Day 14). For comparison, five healthy, uninfected, and untreated mice were used to assess variations in body weight, rectal temperature, and PCV [22, 23]:

%Change in parasitemia Day 140100−=mean parasitemia on Day 140−mean parasitemia on Day mean parasitemia on Day 0×.%Change in body weight Day 140100−=mean body weight on Day 140−mean body weight Day mean body weight on Day 0×.

### 2.12. Homology Modeling and Model Validation Using SWISS‐MODEL

Since the 3D structure of *T. congolense* trypanothione reductase (TR) was not available in the Protein Data Bank (PDB), its structure was predicted using homology modeling through the SWISS‐MODEL server. The FASTA sequence of *T. congolense* TR (UniProt ID P13110) was used as the input for model generation. The sequence was uploaded to the SWISS‐MODEL online workspace (https://www.swissmodel.expasy.org) using the Basic Local Alignment Search Tool (BLAST) (SIB, Lausanne, Switzerland) [25] to generate a homology model. Among the 50 generated templates, the final template selected was the *Trypanosoma brucei* TR structure (PDB ID 6RB5, Chain A) [26], chosen for its highest sequence identity, optimal global model quality estimation (GMQE) and qualitative model energy analysis (QMEAN) scores, extensive query coverage, and superior structural resolution, ensuring maximum accuracy for subsequent 3D model construction.

The quality of the modeled 3D protein structures was evaluated using the QMEAN and GMQE scores, along with Ramachandran plot analysis. A reliable homology model typically exhibits a QMEAN score above −4.0, a GMQE value greater than 0.5, and over 90% of residues in the favored regions of the Ramachandran plot [27]. Based on these assessments, the model with the most favorable quality parameters was selected for subsequent protein preparation steps.

### 2.13. Molecular Docking Study

Subsequently, the molecular docking study of lobetyolin (LR‐1) was performed on the homology‐modeled TR of *T. congolense* using Maestro v.13.5, Schrödinger. The protein structure was first prepared by correcting bond orders, atom types, and charges, removing water molecules beyond 5 Å, and reconstructing any missing side chains and loops. Energy minimization and elimination of steric clashes were carried out using the OPLS4 force field [28]. The ligand, lobetyolin (LR‐1), was drawn in ChemDraw Ultra (2019) and saved in MDL SDfile format. Ligand preparation was then performed in Maestro v13.5 (Schrödinger Suite 2023‐1) using the LigPrep module with the OPLS4 force field at a physiological pH of 7.0 ± 2.0, generating all possible ionization states and stereoisomers [29]. To define the docking site, a receptor grid box was created around the ligand‐binding site with a 6.0 Å radius, adjusting the van der Waals radii with a partial atomic charge scaling factor of 0.8 and a cut off of 0.15 to soften nonpolar regions. Finally, lobetyolin (LR‐1) was docked into the active site using Glide in extra precision (XP) mode. The cocrystallized ligand was redocked first to validate the docking protocol by assessing binding affinity and molecular interactions, and docking scores (kcal/mol) were used to evaluate the predicted binding poses. All procedures were performed using the Schrödinger Suite.

### 2.14. Statistical Analysis

Data analysis was performed using IBM SPSS Statistics for Windows, Version 25.0. Results were presented as mean ± standard error of the mean (M ± SEM). Statistical significance was assessed using one‐way ANOVA followed by the Tukey post hoc test for comparisons between treatment and control groups. A *p* value < 0.05 was considered statistically significant.

## 3. Results

### 3.1. Extraction Yield

In this study, maceration of *L. rhynchopetalum* roots using 80% methanol produced a reddish–brown solid with a yield of 5% by weight (*w*/*w*). Hydroalcoholic extraction was chosen for its effectiveness in extracting a broad range of polar and moderately polar compounds [10]. Further phytochemical investigation of the extract using CC led to the isolation of a pure yellowish solid, designated as LR‐1.

### 3.2. Structural Elucidation of the Isolated Compound (LR‐1)

LR‐1 was obtained as a yellow solid with an *R*_*f*_ value of 0.58 on silica gel TLC using a CHCl_3_/MeOH (4:1) mobile phase. Its molecular formula, C_20_H_28_O_8_, was determined based on pseudomolecular ions that appeared at *m*/*z* = 397.1942 [M + H]^+^ (calcd = 397.1862) and *m*/*z* = 793.3812 [2 M + H]^+^ (calcd = 793.3647) in the high‐resolution positive ion electrospray ionization time‐of‐flight mass spectrum (HR‐ESI‐TOF‐MS).

The ^1^H‐NMR spectrum of compound LR‐1 confirmed the presence of two pairs of *trans*‐coupled double bonds. This was evident from the signals at *δ* 5.65 (*dd*, *J* = 15.5, 1.8 Hz, 1H, H‐3) and *δ* 6.38 (*dq*, *J* = 16.0, 6.9 Hz, 1H, H‐2), as well as *δ* 5.50 (*dd*, *J* = 15.6, 8.0, 1.5 Hz, 1H, H‐10) and *δ* 5.95 (*dt*, *J* = 14.4, 6.9 Hz, 1H, H‐11). As described in the Materials and Methods section, the presence of a glucose moiety in LR‐1 was indicated by its ^1^H‐NMR spectrum. A methine signal was observed at *δ* 4.41 (*J* = 7.8 Hz, H‐1 ^′^), corresponding to the anomeric proton. The large coupling constant (*J* = 7.8 Hz) indicates that the anomeric center adopts a *β*‐configuration [13]. The details of the remaining proton signals are provided in the Materials and Methods section.

**Figure 1:**
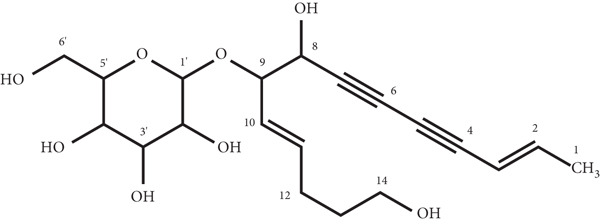
Chemical structure of lobetyolin (LR‐1).

The ^13^C‐NMR spectrum of LR‐1 revealed the presence of 20 carbon atoms, while the DEPT‐135 spectrum identified 16 protonated carbons, consisting of 11 CH, 4 CH_2_, and 1 CH_3_ groups. Additionally, four quaternary carbons were observed at *δ* 69.60 (C‐7), *δ* 71.82 (C‐6), *δ* 76.95 (C‐5), and *δ* 81.25 (C‐4), as these signals were absent in the DEPT spectra. Other important carbon signals included four olefinic carbons (*δ* 109.30, *δ* 144.39, *δ* 125.26, and *δ* 137.29) and two oxymethine carbons (*δ* 65.47 and *δ* 80.70). The presence of the glucose moiety in LR‐1 was also supported by the ^13^C‐NMR spectrum, with four oxymethine carbon signals (*δ* 70.56, *δ* 73.68, *δ* 76.67, and *δ* 77.00), an anomeric carbon (*δ* 99.82), and oxymethylene carbon (*δ* 61.80). The remaining carbon signals are assigned in detail in the Materials and Methods section. Finally, based on data from 1D‐NMR, HRESI‐TOF‐MS, and a comparison of the NMR data with previous studies [13], the isolated compound LR‐1 was unequivocally identified as lobetyolin (Figure 1).

### 3.3. Acute Toxicity

Since toxicity is a major concern in therapeutic preparations originated from medicinal plants [30], ensuring their safety is essential. In this study, the LRE and its constituent lobetyolin showed no signs of toxicity in mice, with no mortality observed within the first 24 h or over the subsequent 14 days. This suggests that the oral LD_50_ exceeds 2000 mg/kg, indicating the extract is safe up to this dosage.

### 3.4. In Vitro Antitrypanosomal Activity of the Extract and Lobetyolin

Parasite motility is a reliable indicator of trypanosome viability [31], and its reduction or elimination compared with the control can serve as a measure of antitrypanosomal activity [32–34]. At maximum test concentration of 4 mg/mL, LRE inhibited parasite motility within 30 min, while DA achieved complete immobility within 20 min at the same concentration (Table 1). All tested LRE concentrations reduced or halted parasite motility, except for the lowest dose (0.1 mg/mL).

**Table 1:** In vitro antitrypanosomal effects of *Lobelia rhynchopetalum* root extract (LRE) and lobetyolin against *Trypanosoma congolense*.

**Dose (mg/mL)**	**Effect**	**1% DMSO**	**DA**	**LRE**	**Lobetyolin**
4.0	Change in motility (in min)	No effect	20^a^	30^a^	25^a^
	No. of infected mice/total no. of mice	5/5	0/5	0/5	0/5
	Infection interval in days (mean ± SEM)	11.60 ± 0.24	No infection	No infection	No infection

2.0	Change in motility (in min)	No effect	30^a^	45^b^	35^a^
	No. of infected mice/total no. of mice	5/5	3/5	3/5	3/5
	Infection interval in days (mean ± SEM)	11.60 ± 0.24	19.0 ± 0.57	16.00 ± 0.57	19.33 ± 0.67

0.4	Change in motility (in min)	No effect	45^b^	65^b^	55^b^
	No. of infected mice/total no. of mice	5/5	4/5	4/5	4/5
	Infection interval in days (mean ± SEM)	11.60 ± 0.24	17.0 ± 0.57	14.75 ± 0.47	16.50 ± 0.5

0.1	Change in motility (in min)	No effect	No effect	No effect	No effect
	No. of infected mice/total no. of mice	5/5	5/5	5/5	5/5
	Infection interval in days (mean ± SEM)	11.60 ± 0.24	12.2 ± 0.37	11.8 ± 0.24	12.60 ± 0.245

*Note:* 1% DMSO (dimethyl sulfoxide): vehicle; LRE: 80% methanolic extract.

Abbreviation: DA, diminazene aceturate.

^a^Motility ceased.

^b^Motility drastically reduced.

Lobetyolin inhibited the motility of the parasites in a dose‐dependent manner. At a concentration of 4 mg/mL, it completely immobilized the parasites within 25 min, while at 2 mg/mL, complete immobility was achieved within 35 min. In comparison, the reference compound DA induced parasite immobility approximately 5 min earlier than lobetyolin at both concentrations (4 and 2 mg/mL). Furthermore, lobetyolin significantly reduced parasite motility even at a lower concentration of 0.4 mg/mL, with noticeable inhibition observed within 55 min (Table 1). However, parasite immobility does not always indicate mortality, as they may remain viable but lose infectivity [15].

In the in vivo infectivity test, all mice that received blood infected with parasites and subsequently incubated with 4 mg/mL of either lobetyolin or DA remained free of infection, indicating complete suppression of parasite infectivity at this concentration. In contrast, all previously uninfected mice developed parasitemia by Day 12 after being injected with infected blood that had been incubated with 0.1 mg/mL of either lobetyolin or DA, demonstrating that this lower concentration was insufficient to prevent infection (Table 1).

### 3.5. In Vivo Infectivity of LRE and Lobetyolin

The blood incubation assay assessed parasite survival in mice over 30 days after intraperitoneal inoculation. DA at 4 mg/mL completely cleared infection, while 2 and 0.4 mg/mL produced partial protection, and full infection occurred at 0.1 mg/mL. The LRE showed a similar pattern: 4 mg/mL prevented infection, intermediate doses gave partial protection, and all mice were infected at 0.1 mg/mL, though infection appeared later than in controls. Lobetyolin at 4 mg/mL also kept mice parasite‐free for the full observation period, comparable with DA. Lower doses of both DA and lobetyolin delayed, but did not prevent, the onset of parasitemia (Table 2).

**Table 2:** Blood incubation results of the 80% methanol extract of *L. rhynchopetalum* (LRE) and lobetyolin.

**Test substance**	**Test concentration**	**Number of mice that developed infection**	**Infection intervals in days (** **m** **e** **a** **n** ± **S** **E** **M** **)**
Lobetyolin	4.0 mg/mL	0/5	Ni
	2.0 mg/mL	3/5	19.33 ± 0.67
	0.4 mg/mL	4/5	16.50 ± 0.50
	0.1 mg/mL	5/5	12.60 ± 0.25

LRE	4.0 mg/mL	0/5	Ni
	2.0 mg/mL	3/5	16.00 ± 0.57
	0.4 mg/mL	4/5	14.75 ± 0.47
	0.1 mg/mL	5/5	11.80 ± 0.24

DA	4.0 mg/mL	0/5	Ni
	2.0 mg/mL	3/5	19.00 ± 0.57
	0.4 mg/mL	4/5	17.00 ± 0.57
	0.1 mg/mL	5/5	12.2 ± 0.37

1% DMSO	0.1 mL	5/5	11.60 ± 0.24

*Note:* Values are mean ± SEM; *N* = 5.

Abbreviations: DA, diminazene aceturate; DMSO, dimethyl sulfoxide; LRE, 80% methanol root extract of *Lobelia rhynchopetalum*; Ni, no infection.

### 3.6. In Vivo Antitrypanosomal Activity of the Extract and Lobetyolin

**Figure 2:**
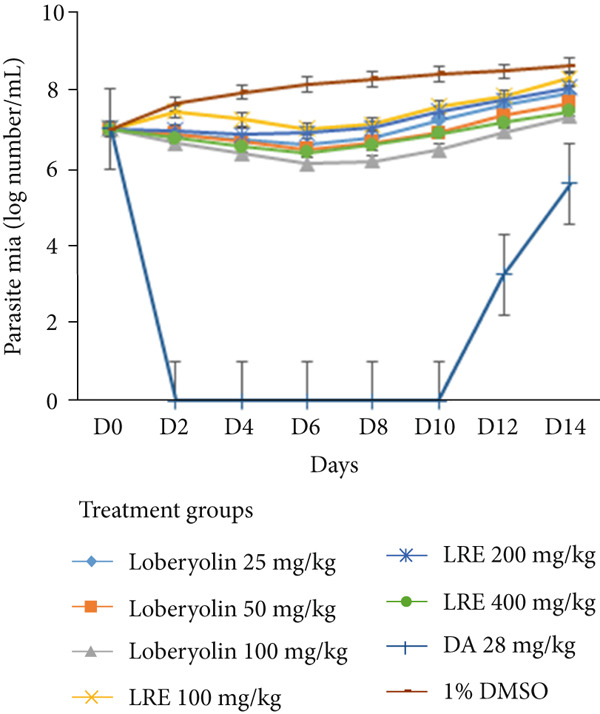
The effect of 80% methanol extract (LRE) and lobetyolin on parasitemia level of *Trypanosoma congolense*–infected mice.

The hydroalcoholic extract, LRE, along with the isolated compound lobetyolin, was further evaluated for their in vivo antitrypanosomal activities in order to gain deeper insights into their therapeutic potential. Indeed, infected mice treated with LRE at daily oral doses of 400 and 200 mg/kg exhibited a significant reduction in parasitemia levels beginning on Day 2 posttreatment, with the suppression continuing through Day 6. However, after Day 6, parasitemia levels began to rise again, indicating a temporary effect of the treatment (Figure 2 and Supporting Information 1).

A similar pattern was observed with lobetyolin across all tested doses. Notably, treatment with lobetyolin at a dose of 100 mg/kg/day resulted in a more pronounced and sustained suppression of parasitemia compared with the other lobetyolin doses, the LRE‐treated groups, and the negative control. This suggests that 100 mg/kg/day of lobetyolin exhibits the most potent in vivo antitrypanosomal activity among the tested regimens.

Mice treated with lobetyolin at a dose of 100 mg/kg/day showed a significantly lower mean parasitemia level (7.320 ± 0.080) on Day 14 posttreatment compared with the negative control group (8.620 ± 0.037), as well as compared with all other lobetyolin and LRE treatment groups. At this dose, the change in parasitemia from the pretreatment level on Day 14 was only 4.57%, indicating strong suppression of parasite growth. Similarly, treatment with LRE at a dose of 400 mg/kg/day resulted in an 11.14% increase in parasitemia on Day 14 compared with the pretreatment level, whereas the negative control group showed a 23.4% increase over the same period. The relapse seen in the DA‐treated group is consistent with previous reports of reduced drug sensitivity in field isolates of *T. congolense* and the drug′s relatively short persistence in plasma, which can allow some parasites to survive and reestablish infection [35, 36]. In addition, parasites may hide temporarily in tissue compartments and later reappear in the bloodstream, further contributing to posttreatment relapse [37] (Supporting Information 1).

### 3.7. Effects of LRE and Lobetyolin on PCV, Body Weight, and Rectal Temperature of *T. congolense*‐Infected Mice

Trypanosomiasis is commonly associated with anemia and reduced appetite, both of which can negatively affect the PCV of affected animals [38]. An improvement in PCV following treatment may indicate either a reduction in parasitemia or the alleviation of the toxic effects of trypanosome‐derived metabolites [16]. Among the treatment groups, mice treated with 100 mg/kg/day of lobetyolin and 400 mg/kg/day of LRE showed the smallest difference in PCV values between Day 0 and Day 14. Notably, in the group treated with 100 mg/kg/day of lobetyolin, the PCV values remained within the normal reference range (48.78 ± 0.48) (Table 3).

**Table 3:** The effect of 80% methanol extract and lobetyolin on PCV of *Trypanosoma congolense*–infected mice.

**Days**	**1% DMSO**	**Lobetyolin** **25 mg/kg**	**Lobetyolin** **50 mg/kg**	**Lobetyolin** **100 mg/kg**	**LRE** **100 mg/kg**	**LRE** **200 mg/kg**	**LRE** **400 mg/kg**	**DA** **28 mg/kg**
Preinfection	58.18 ± 0.72	60.52 ± 1.03	59.94 ± 1.00	59.76 ± 0.92	61.68 ± 0.30	61.18 ± 0.34	60.26 ± 0.57	60.34 ± 0.31
Day 0	48.38 ± 0.51^a^	49.88 ± 0.26^b^	50.14 ± 0.28^b,c^	50.06 ± 0.41^c^	50.52 ± 0.21^c^	50.40 ± 0.15^c^	49.66 ± 0.25^b^	50.12 ± 0.27^c^
Day 7	43.62 ± 0.58^a^	46.82 ± 0.32^b^	48.72 ± 0.44^c^	49.38 ± 0.45^c,d^	46.48 ± 0.19^b^	47.50 ± 0.14^c^	48.64 ± 0.25^c^	51.22 ± 0.30^d^
%Change PCV (Day 7−0)	−10.94	−6.54	−2.93	−1.38	−8.69	−6.11	−2.1	2.15
Day 14	38.58 ± 0.83^a^	44.96 ± 0.24^b^	47.88 ± 0.52^c^	48.78 ± 0.48^c^	43.70 ± 0.29^b^	45.70 ± 0.13^b^	48.00 ± 0.39^c^	51.08 ± 0.43^d^
%Change PCV (Day 14−7)	−13.16	−4.14	−1.76	−1.23	−6.37	−3.94	−1.34	−0.31

*Note:* Data are presented as mean ± SEM (*n* = 5). Different letters within the same row indicate significant differences (*p* < 0.05).

Abbreviation: LRE, 80% methanol root extract of *Lobelia rhynchopetalum*.

Body weight changes can be influenced by multiple factors. Weight loss typically results from a combination of reduced food intake and increased energy demands. In trypanosome infections, symptoms such as fever, appetite loss, and anemia contribute to this effect. Appetite suppression is partly attributed to the release of interleukin‐1, which affects the hypothalamus and may also influence gut motility. Additionally, reduced oxygen delivery due to decreased PCV is another factor contributing to weight loss [15].

Mice treated with lobetyolin at a dose of 100 mg/kg/day exhibited a significant (*p* < 0.05) increase in body weight—approximately 4%—compared with those treated with LRE (at the same dose) and the negative control group. This weight gain was comparable with that observed in the positive control group, with no statistically significant difference between them (Table 4 and Supporting Information 2). The observed improvement in body weight may be attributed to the favorable PCV profile, suggesting reduced red blood cell hemolysis, more effective parasite suppression at this dose, and improved appetite in the treated mice—findings consistent with previous studies [23, 39].

**Table 4:** The effects of 80% methanol extract of *L. rhynchopetalum* and lobetyolin on the body weight.

**Group**	**1% DMSO**	**Lobetyolin** **25 mg/mg**	**Lobetyolin** **50 mg/mg**	**Lobetyolin** **100 mg/kg**	**LRE** **100 mg/kg**	**LRE** **200 mg/kg**	**LRE** **400 mg/kg**	**DA** **28 mg/kg**
Preinfection BW	22.98 ± 0.42	23.48 ± 0.59	23.08 ± 0.79	23.94 ± 0.84	24.00 ± 0.88	23.06 ± 0.20	23.36 ± 0.85	23.52 ± 0.54
Day 0	21.08 ± 0.35	20.84 ± 0.34	21.58 ± 0.77	22.00 ± 0.67	20.34 ± 0.19	21.26 ± 0.07	21.76 ± 0.55	22.38 ± 0.38
Day 7	19.74 ± 0.21^a^	20.94 ± 0.22^b^	21.82 ± 0.76^b,c^	22.36 ± 0.64^c^	20.32 ± 0.19^b^	21.42 ± 0.09^b^	21.92 ± 0.56^c^	22.62 ± 0.37^c^
Day 14	18.74 ± 0.25^a^	21.12 ± 0.22^b^	21.98 ± 0.74^b^	22.88 ± 0.60^c^	20.44 ± 0.19^d^	21.56 ± 0.06^b^	22.16 ± 0.53^c^	23.40 ± 0.23^e^
%Change BW (Day 14−0)	−11.10	1.34	1.85	4.00	0.49	1.41	1.84	4.56

*Note:* Data are presented as mean ± SEM (*n* = 5). Different letters within the same row indicate significant differences (*p* < 0.05).

Abbreviation: LRE, 80% methanol root extract of *Lobelia rhynchopetalum*.

Pyrexia typically occurs in cycles, corresponding with waves of parasitemia that emerge after the onset of infection. It is also associated with increased energy demands for physiological maintenance [23]. Both LRE and lobetyolin significantly reduced the rectal temperature of infected mice by Day 14 posttreatment compared with their respective pretreatment values (Table 5 and Supporting Information 3).

**Table 5:** The effects of 80% methanol extract of *L. rhynchopetalum* and lobetyolin on the rectal temperature.

**Days**	**1% DMSO**	**Lobetyolin** **25 mg/kg**	**Lobetyolin** **50 mg/kg**	**Lobetyolin** **100 mg/kg**	**LRE** **100 mg/kg**	**LRE** **200 mg/kg**	**LRE** **400 mg/kg**	**DA** **28 mg/kg**
Preinfection	35.74 ± 0.20	34.84 ± 0.34	35.8 ± 0.08	33.32 ± 0.52	36.02 ± 0.34	35.74 ± 0.22	35.16 ± 0.22	34.08 ± 0.32
Day 0	38.78 ± 0.24^a^	36.52 ± 0.81^b^	38.72 ± 0.31^a^	38.92 ± 0.23^a^	38.60 ± 0.19^a^	38.48 ± 0.19^a^	39.26 ± 0.31^c^	38.8 ± 0.49^a^
Day 7	39.5 ± 0.30^da^	36.14 ± 0.19^b^	36.24 ± 0.34^b^	34.48 ± 0.48^c^	37.92 ± 0.15^d^	36.7 ± 0.31^b^	36.08 ± 0.06^b^	34.4 ± 0.52^c^
Day 14	40.62 ± 0.19^a^	37.24 ± 0.39^b^	37.82 ± 0.18^b^	35.98 ± 0.24^c^	38.74 ± 0.08^d^	37.66 ± 0.33^b^	37.2 ± 0.28^b^	35.48 ± 0.15^c^

*Note:* Data are presented as mean ± SEM (*n* = 5). Different letters within the same row indicate significant differences (*p* < 0.05).

Abbreviation: LRE, 80% methanol root extract of *Lobelia rhynchopetalum*.

### 3.8. Homology Model

Since the crystal structure of *T. congolense* TR is not available in the PDB database, we constructed a structural model through homology modeling. The TR structure from *T. brucei* (PDB ID 6RB5, Chain A) was used as the reference template. The model was built using the SWISS‐MODEL server, which produced a GMQE value of 0.93 and a QMEAN score of 0.87. Both parameters suggest that the predicted structure is reliable, as GMQE values close to 1.0 indicate greater accuracy and QMEAN values above −4.0 are considered acceptable for structural quality. In addition, Ramachandran plot evaluation showed that approximately 96.81% of the amino acid residues fall within the favored regions. Taken together, these results indicate that the modeled TR structure is stable and provides a good representation of the likely native form. Figure 3 depicts the graphical representation of GMQE, QMEAN, and Ramachandran plots of homology modeled TR structure.

Figure 3Ramachandran plot (a) and QMEANDisCo (b) of homology‐modeled TR structure.

**Figure:**
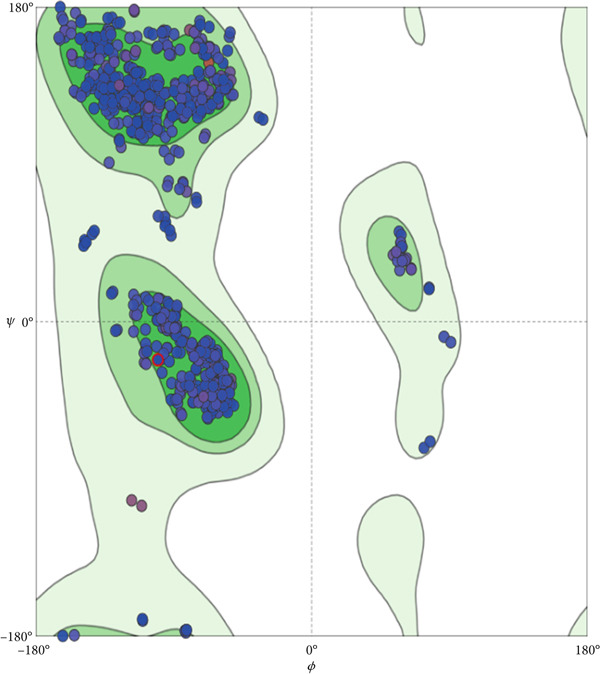
(a)

**Figure:**
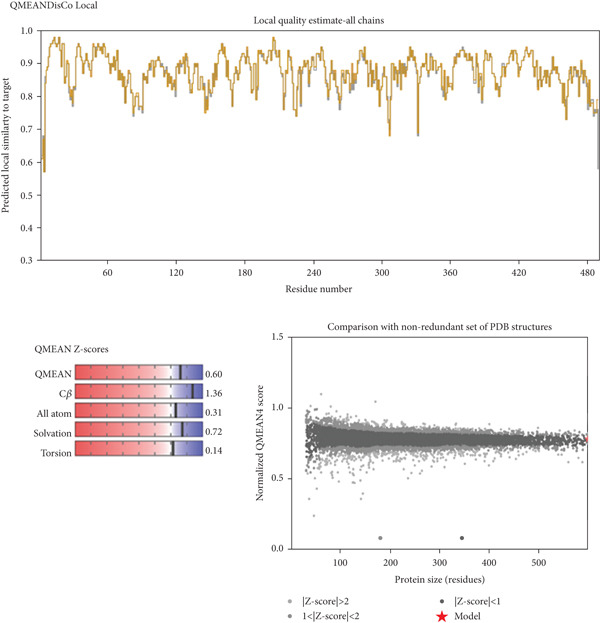
(b)

### 3.9. Molecular Docking Analysis

Molecular docking predicts how small molecule drug candidates bind to their protein targets, helping to assess their affinity and potential activity [40, 41]. Key antitrypanosomal targets include TR, trypanothione synthetase (TryS), glyceraldehyde‐3‐phosphate dehydrogenase (GAPDH), sterol 14*α*‐demethylase (CYP51), and cysteine proteases. Quick screening revealed that TR interacts favorably with lobetyolin.

The docking study showed that lobetyolin binds strongly to the homology‐modeled TR from *T. congolense*. Its Glide score was −8.002 kcal/mol, which is close to that of the enzyme′s native ligand (−8.307 kcal/mol), suggesting a comparable binding strength (Table 6). In the binding pocket, lobetyolin formed hydrogen bonds with GLU A:18, GLU B:466, SER A:14, and TYR A:110, interactions that appear to play an important role in stabilizing the ligand. The compound also engaged in several hydrophobic and van der Waals contacts with residues including ILE A:106, VAL A:53 and 58, MET B:400, LEU A:62 and B:399, and TRP A:21, which contribute additional stabilization to the complex. Three‐dimensional visualization further confirmed a well‐fitted orientation of lobetyolin within the catalytic site, interacting with residues on both Chain A and Chain B.

**Table 6:** Docking scores of lobetyolin and native ligand within the active site of TR.

**Ligand**	**Glide score (kcal/mol) within TR**	**Interaction with amino acid residues**	
		**H-bonds**	**Non-H-bonds**
Lobetyolin	−8.002	GLU A:18; GLU B:466; SER A:14; TYR A:110	ILE A:106; VAL A:53; VAL A:58; MET B:400; LEU B:399; LEU A:62; PRO B:462; PRO A:366; ILE A:339; TRP A:21; LEU A:17; HIS B:461; THR A:335; SER B:470
Native ligand	−8.307	GLU A:18; GLY A:49; GLY A:13; SER A:109	MET A:113; TYR A:110; ILE A:106; LEU B:399; PHE B:396; VAL A:53; ILE A:399; LEU A:17; TRP A:21; SER A:109; SER A:14

*Note:* Native ligand = 4‐(((3‐(8‐(2‐((1R,2S,5R)‐6,6‐dimethylbicyclo[3.1.1]heptan‐2‐yl)ethyl)‐4‐oxo‐1‐phenyl‐1,3,8‐triazaspiro[4.5]decan‐3‐yl)propyl)(methyl)amino)methyl)‐4‐hydroxypiperidine‐1‐carboximidamide.

These results indicate that lobetyolin can interact strongly with TR and may act as an inhibitor of the enzyme. By occupying the active site, the compound could interfere with the enzyme′s catalytic function and, in turn, disturb the redox balance that is vital for the parasite′s survival. Figure 4 illustrates both the 2D and 3D representations of lobetyolin bound within the active site of the modeled TR enzyme, showing its orientation in the pocket along with the key residues involved in the interaction.

Figure 4Interaction of lobetyolin with the homology‐modeled trypanothione reductase (TR) from *Trypanosoma congolense*. (a) 2D interaction map showing key contacts between the ligand and active‐site residues. (b) 3D view of the ligand positioned within the catalytic pocket, visualized using Schrödinger Maestro.

**Figure:**
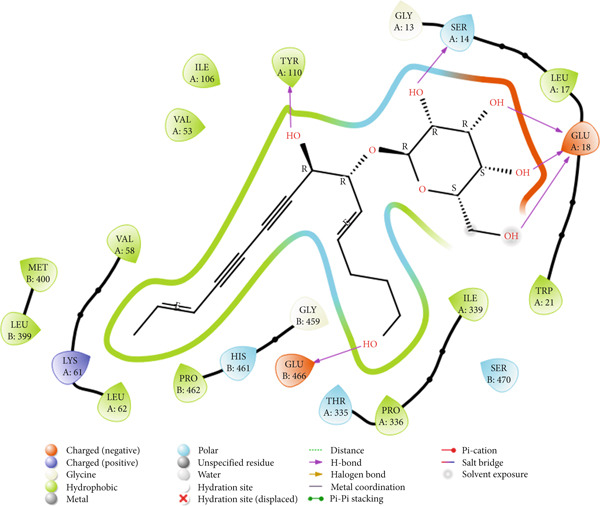
(a)

**Figure:**
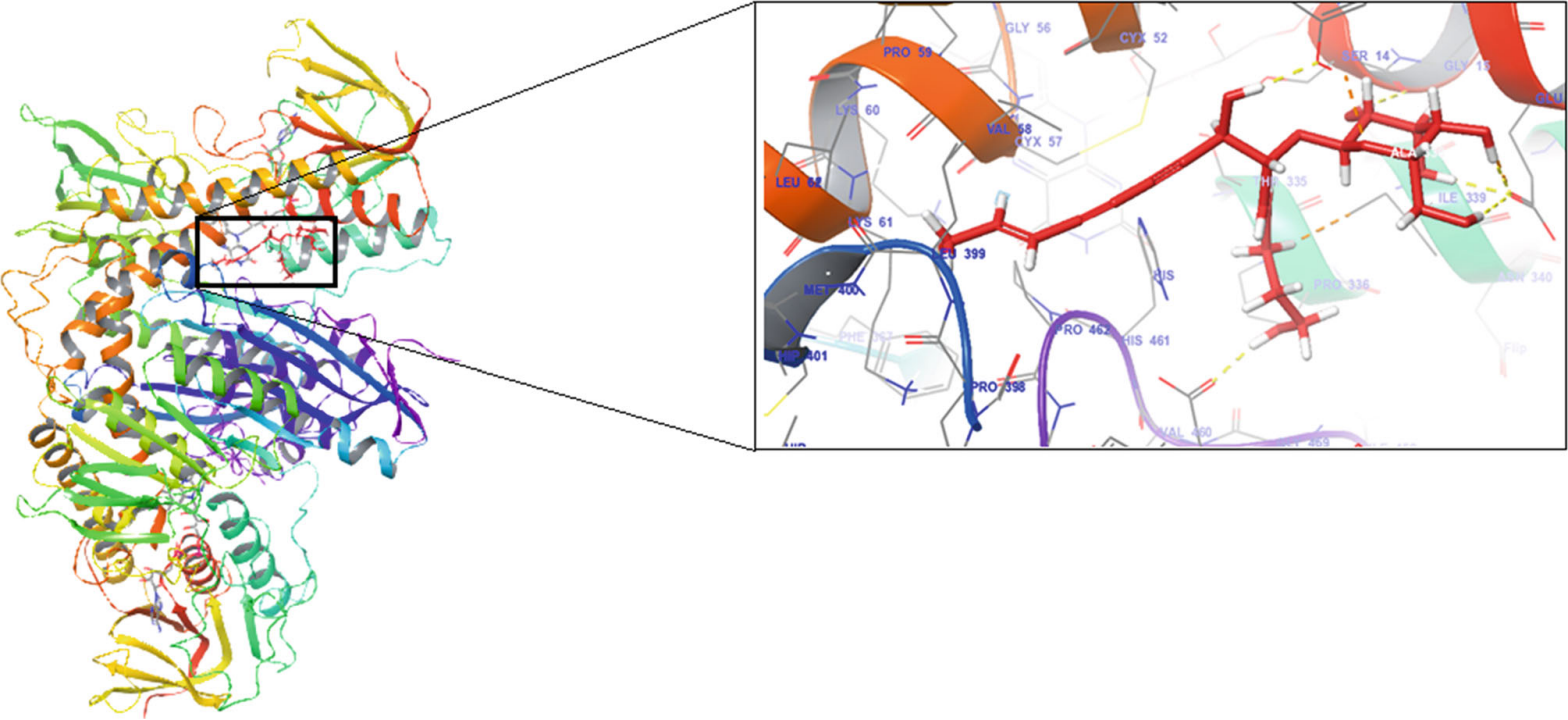
(b)

To validate the reliability of the docking procedure, the native ligand was redocked into the active site of the modeled *T. congolense* TR (Supporting Information 4). The native ligand produced a Glide score of −8.307 kcal/mol and maintained key hydrogen bonding and hydrophobic contacts with residues within the catalytic pocket (Table 6). Its binding pose was consistent with the expected orientation in the active site. The close agreement between the binding score and interaction profile of the native ligand and those observed for lobetyolin supports the validity of the docking protocol and indicates that the results are both accurate and biologically meaningful.

In summary, lobetyolin may inhibit TR by interfering with its catalytic activity, thereby disrupting the parasite′s redox homeostasis. However, further research is needed to fully understand its interaction with TR and its potential as an antiparasitic agent.

## 4. Discussion

Several traditional medicinal plants have previously been validated for their antitrypanosomal activity, and their active compounds have been isolated [42, 43]. This study examined *L. rhynchopetalum*, a plant traditionally used in Ethiopia to treat protozoal diseases [9, 10], and confirmed its antitrypanosomal activity through both in vitro and in vivo models. Additionally, the study identified lobetyolin as a major constituent from the active extract and performed molecular docking on TR to investigate its mechanism of antitrypanosomal action.

The highest dose (4 mg/mL) of the extract showed strong antitrypanosomal activity, likely by disrupting critical metabolic pathways. Although lower concentrations may not completely immobilize the parasites, they could still impair their ability to infect mice. This may occur because the extract and lobetyolin alter the parasites′ morphology, making them more vulnerable to the mice′s immune response, or because they interfere with essential metabolic processes [23].

Lobetyolin, a polyacetylene, is a class of compounds characterized by alternating single and triple bonds in their carbon backbone. Polyacetylenes have been investigated for their antiparasitic properties. For instance, a study published by Greger [44] reviewed the biological activities of polyacetylenes found in the genus *Artemisia*, highlighting their antifungal, insecticidal, nematicidal, and cytotoxic properties. Additionally, studies have identified polyacetylenes containing a reactive triple bond capable of readily alkylating SH groups. For example, Carlina oxide from *Carlina acaulis* (Asteraceae) and polyacetylenes from ginseng (*Panax ginseng*) exhibit significant cytotoxic activity against *T. brucei brucei* while showing minimal toxicity to human cells [45, 46]. Trypanosomes are highly sensitive to ergosterol biosynthesis inhibitors, and specific sterols are very crucial for the viability of trypanosomes. Polyacetylenes have been shown to inhibit the cholesterol acyltransferase; therefore, this is probably the other mechanism of lobetyolin′s antitrypanosomal activity [46].

The docking analysis indicates that lobetyolin and the native ligand show almost the same binding affinity toward the homology‐modeled TR from *T. congolense*. Their Glide scores (−8.002 and −8.307 kcal·mol^−1^, respectively) differ by only 0.305 kcal·mol^−1^, which is within the expected margin of error for docking studies. Both molecules occupy similar regions in the enzyme′s active site and share common interaction patterns. Notably, lobetyolin forms additional contacts with residues on both Chain A and Chain B, suggesting that it may engage a slightly broader binding interface, potentially supporting a competitive inhibition mechanism.

However, as docking provides a static view and is based on a predicted structure, these results should be interpreted with caution. Experimental validation, such as differential scanning fluorimetry (DSF), surface plasmon resonance (SPR), or isothermal titration calorimetry (ITC), would be necessary to confirm the binding behavior and inhibitory potential of lobetyolin. Since TR plays a crucial role in maintaining the parasite′s redox balance by regulating oxidative stress, its inhibition could lead to an accumulation of reactive oxygen species (ROS), ultimately disrupting the parasite′s redox homeostasis and impairing its survival. Oxidative stress in trypanosome infections occurs when the production of ROS exceeds the parasite′s ability to neutralize them or repair the resulting damage. Maintaining a reducing environment is essential for the parasite′s survival, and this balance is sustained by enzymes that continuously provide metabolic energy to keep the system in a reduced state. Disruptions to this redox balance can lead to toxicity, as the accumulation of peroxides and free radicals can damage critical cellular components, including proteins and lipids. Many natural compounds also exhibit trypanocidal activity by disrupting the parasite′s redox homeostasis, either by targeting the respiratory chain or interfering with cellular defenses against oxidative stress [17]. Although lobetyolin is known to act as an antioxidant in mammalian cells [47], its effect in *Trypanosoma* appears to work differently. The parasite relies on the trypanothione system, and specifically the enzyme TR, to keep trypanothione in its reduced form so it can neutralize ROS. When TR is inhibited, this protective mechanism weakens, ROS starts to build up, and the parasite experiences oxidative stress and a breakdown in redox balance, which can ultimately compromise its survival [48]. In the end, lobetyolin, a bioactive compound extracted from the roots of *L. rhynchopetalum*, shows significant potential as a therapeutic agent against trypanosomiasis. These findings suggest that lobetyolin could serve as a valuable lead compound for the development of new antitrypanosomal treatments and highlight its promise for further investigation.

## 5. Conclusion

This study provides the first evidence that the LRE and its major constituent, lobetyolin, possess antitrypanosomal activity. The observed effect of the extract may be largely due to lobetyolin, which is also suggested to inhibit the TR enzyme, potentially disrupting the parasite′s redox balance and leading to its death. These results support the traditional use of the plant against parasitic infections and, for the first time, reveal the antitrypanosomal potential of both *L. rhynchopetalum* and lobetyolin, highlighting the need for further investigation.

## Ethics Statement

All applicable international, national, and institutional guidelines for the care and use of laboratory animals were strictly followed. The experiments were conducted in accordance with internationally accepted standards for the use and care of laboratory animals [11, 12]. All animal procedures were approved by and carried out in compliance with the ethical standards of the School of Pharmacy, College of Health Sciences, Addis Ababa University (Protocol No. ERB/SOP/417/14/2022).

## Disclosure

All authors reviewed and approved the final version of the manuscript.

## Conflicts of Interest

The authors declare no conflicts of interest.

## Author Contributions

S.Y.: conceived and designed the experiments, conducted the investigation and data analysis, and wrote the original draft of the manuscript. E.T.: assisted with the evaluation of biological activity. D.B.: performed the molecular docking analysis and contributed to manuscript editing. S.T.: contributed to experimental design, provided supervision, secured funding, and participated in writing and editing the manuscript. M.Y.Y.: participated in experimental design and supervision and contributed to manuscript editing.

## Funding

This study was supported by the International Science Program, Uppsala University (ETH:02), Addis Ababa University (10.13039/501100007941), and Bahir Dar University (10.13039/501100005872).

## Supporting information


**Supporting Information** Additional supporting information can be found online in the Supporting Information section. Supporting Information 1 TLC chromatograms of the 80% methanol extract of *Lobelia rhynchopetalum.* Supporting Information 2 LC‐MS of lobetyolin. Supporting Information 3 ^1^H NMR of lobetyolin. Supporting Information 4 ^13^C NMR of lobetyolin. Supporting Information 5 DEPT‐135 of lobetyolin. Supporting Information 6 ^1^H^1^H‐COSY of lobetyolin. Supporting Information 7 HMBC of lobetyolin. Supporting Information 8 HSQC of lobetyolin. Supporting Information 9 ^1^H, ^13^C, and 2D NMR data of lobetyolin measured in acetone‐D6. Supporting Information 10 The effects of LRE and lobetyolin on body weight of *Trypanosoma congolense*–infected mice. Supporting Information 11 Effects of LRE and lobetyolin on body weight of *Trypanosoma congolense*–infected mice. Supporting Information 12 Effects of LRE and lobetyolin on rectal temperature of *Trypanosoma congolense*–infected mice.

## Data Availability

All data and materials supporting the results are presented in the manuscript.
