# Supplementary material for: Antitrypanosomal Activity and Molecular Docking Studies of Lobetyolin From Lobelia rhynchopetalum Hemsl. Root Extract Against Trypanosoma congolense Field Isolates
Source: Biomed Res Int. 2026 Feb 4;2026:2214310. doi: 10.1155/bmri/2214310 (PMC12872960; doi:10.1155/bmri/2214310)
Supplement: Supplementary file 1 — Supporting Information Additional supporting information can be found online in the Supporting Information section. Supporting Information 1 TLC chromatograms of the 80% methanol extract of Lobelia rhynchopetalum. Supporting Information 2 LC‐MS of lobetyolin. Supporting Information 3 1H NMR of lobetyolin. Supporting Information 4 13C NMR of lobetyolin. Supporting Information 5 DEPT‐135 of lobetyolin. Supporting Information 6 1H1H‐COSY of lobetyolin. Supporting Information 7 HMBC of lobetyolin. Supporting Information 8 HSQC of lobetyolin. Supporting Information 9 1H, 13C, and 2D NMR data of lobetyolin measured in acetone‐D6. Supporting Information 10 The effects of LRE and lobetyolin on body weight of Trypanosoma congolense–infected mice. Supporting Information 11 Effects of LRE and lobetyolin on body weight of Trypanosoma congolense–infected mice. Supporting Information 12 Effects of LRE and lobetyolin on rectal temperature of Trypanosoma congolense–infected mice. [file BMRI-2026-2214310-s001.docx]

**Supporting Information**

**Antitrypanosomal Activity and Molecular Docking Studies of Lobetyolin from *Lobelia rhynchopetalum* Hemsl. Root Extract against *Trypanosoma congolense* Field Isolates**

**Selamawit Yimer,^1,2^ Eyael Tewelde,^2^ Daniel Bisrat,^2^ Solomon Tadesse,^3^ and Mariamawit Y. Yeshak^2^***

*^1^Department of Pharmacognosy, School of Pharmacy, College Of Health Sciences, Bahir Dar University, P.O. Box 79, Bahdir Dar, Ethiopia*

*^2^Department of Pharmaceutical Chemistry and Pharmacognosy, School of Pharmacy, College of Health Sciences, Addis Ababa University, P.O. Box 1176, Addis Ababa, Ethiopia;*

*^3^Department of Biomedical and Pharmaceutical Sciences, College of Pharmacy, Idaho State University, 921 South 8th Avenue, Stop 8333, Pocatello, ID 83209-8333, Idaho, 83209, USA*

*Corresponding author: Mariamawit Y. Yeshak; Tel: +251911506609; E-mail: mariamawit.yonathan@aau.edu.et

Selamawit Yimer: e-mail: selamy67@gmail.com

Eyael Tewelde: e-mail: eyaeltd@gmail.com

Daniel Bisrat: e-mail: daniel.bisrat@aau.edu.et

Solomon Tadesse: e-mail: [solomonzeleke@isu.edu](mailto:solomonzeleke@isu.edu), <https://orcid.org/0000-0002-9966-2236>

Mariamawit Y. Yeshak: e-mail: [mariamawit.yonathan@aau.edu.et](mailto:mariamawit.yonathan@aau.edu.et), <https://orcid.org/0000-0003-0918-3635>


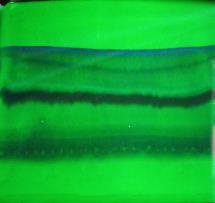

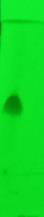


PTLC and CC

Supporting Information 1: TLC chromatograms of the 80% methanol extract of *Lobelia rhynchopetalum.*


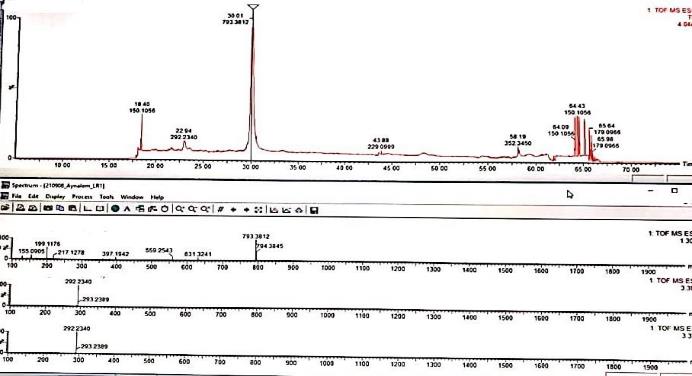


Supporting Information 2: LC-MS of lobetyolin.


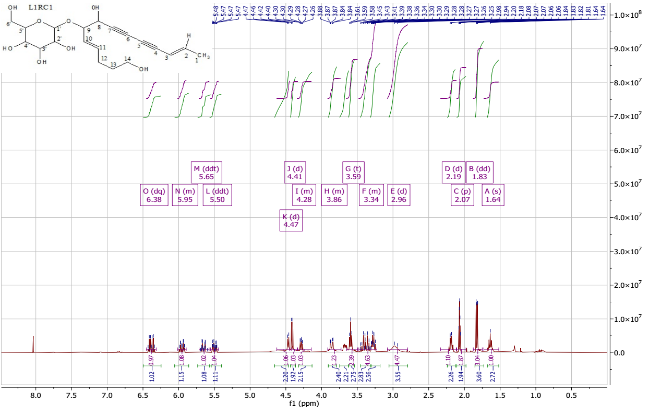


**Supporting Information 3: ^1^H NMR of lobetyolin.**


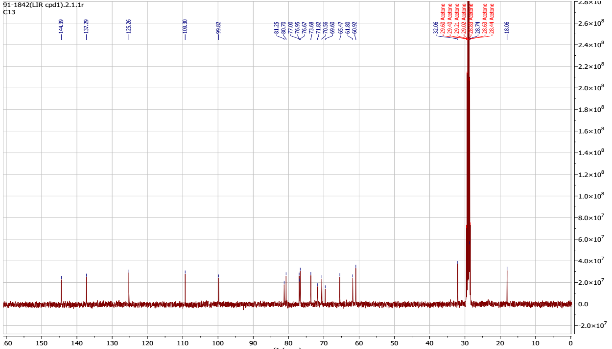


Supporting Information 4: ^13^C NMR of lobetyolin.


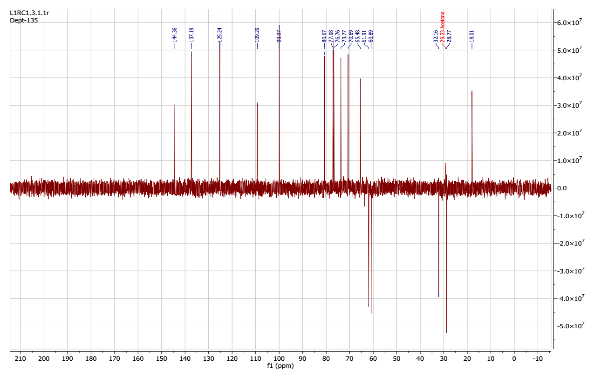


Supporting Information 5: DEPT-135 of lobetyolin.


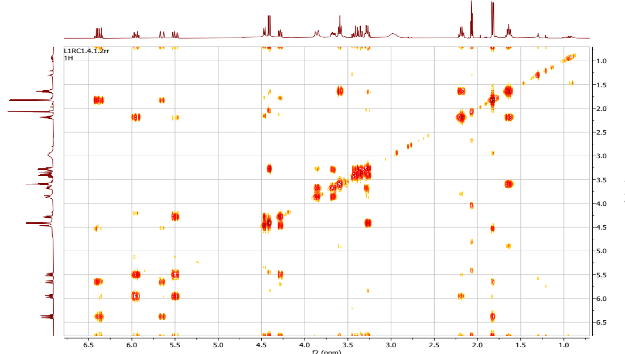

**Supporting Information 6: ^1^H^1^H-COSY of lobetyolin.**


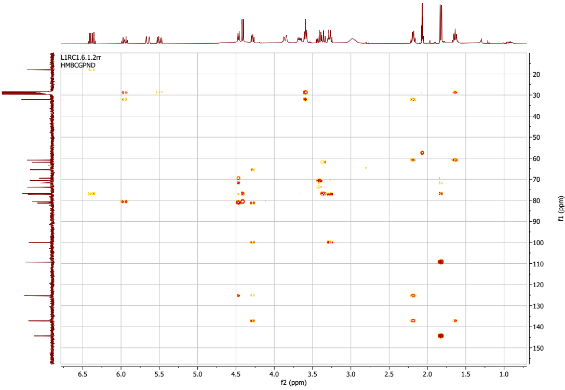

##

## **Supporting Information 7**: **HMBC of lobetyolin.**


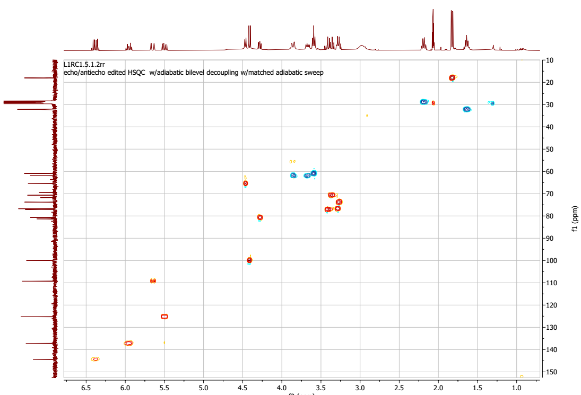

**Supporting Information 8: HSQC of lobetyolin.**

**Supporting Information 9**: **^1^H, ^13^C and 2D NMR data of lobetyolin measured in acetone-D6.**

| **C position** | **Present data** | | | | | **Reference (Ishimaru *et al*., 1991)** | |
| --- | --- | --- | --- | --- | --- | --- | --- |
|  | **^1^H NMR δ (ppm)** | **^13^C NMR δ (ppm)** | **COSY (H-H)** | **HSQC**  **(H-C)** | **HMBC**  **H-C** | **^1^H NMR lobetyolin δ** | **^13^C NMR Lobetyolin δ (ppm)** |
|  |  |  |  |  |  | **(ppm)** |  |
| 1 | 1.83 (dd, *J* = 6.9, 1.9 Hz, 3H) | 18.06 | 6.38-1.83 | 1.83-18.06 | 1.83-18.06 | 1.73 (dd, *J*=6.8, 2.3 Hz, 3H) | 19.20 |
| 2 | 6.38 (dq, *J* = 16.0, 6.9 Hz, 1H) | 144.39 | 6.38-5.65 | 6.38-144.39 | 6.38-1.83 | 6.29 (dq, *J*=16.2, 6.8, 1H) | 145.80 |
| 3 | 5.65 (dd, *J* = 15.5, 1.8 Hz, 1H | 109.30 | 6.38-5.65 | 5.65-109.30 |  | 5.55 (dd, *J*=16.2, 2.3, 1H) | 110.40 |
| 4 | - | 81.25 | - | - |  | - | 82.40 |
| 5 | - | 76.95 | - | - |  | - | 78.20 |
| 6 | - | 71.82 | - | - |  | - | 72.90 |
| 7 | - | 69.60 | - | - |  | - | 70.80 |
| 8 | 4.47 (d, *J* = 6.1 Hz, 1H) | 65.47 | 4.47-4.28 | 4.47-65.47 | 4.47-80.70 | 4.40 (d, *J*=6.8 Hz,1H) | 66.50 |
| 9 | 4.28 (m, *J* = 7.9, 6.0 Hz, 1H | 80.70 | 4.28-5.50 | 4.28-80.70 | 4.28-137.29, 4.28-99.82 | 4.20 (t, *J*= 6.8 Hz, 1H) | 81.80 |
| 10 | 5.50 (dd, *J* = 15.6, 8.0, 1.5 Hz, 1H), | 125.26 |  | 5.50-125.26 | 5.50-28.74 | 5.40 (dd, *J*=16.2, 6.8 Hz, 1H) | 126.40 |
| 11 | 5.95 (dt, *J* = 14.4, 6.9 Hz, 1H) | 137.29 | 5.94-5.50,5.95-2.19 | 5.95-137.29 | 5.95-80.700, 5.95-28.74,  5.95-32.06 | 5.86 (dt, *J*= 16.2, 6.8 Hz, 1H) | 138.6 |
| 12 | 2.19 (dd, *J* = 7.5 Hz, 2H) | 28.74 | 2.19-1.64 | 2.19-28.74 | 2.19-137.29 & 2.19-125.26 | 2.09 (*br* dd, *J*=13.5, 6.8, 2H) | 29.70 |
| 13 | 1.64 (q, *J* = 6.9 Hz, 2H) | 32.06 | 1.64-3.59 | 1.64-32.06 | 1.64-137.29, 1.64-60.92,1.64-28.74 | 1.56(quin, *J*=6.8, 2H) | 33.00 |
| 14 | 3.59 (m,2H) | 60.92 | 3.59-1.64 | 3.59-60.92 | 3.59-32.06,3.59-28.74 | 3.49 (t, 2H, *J*= 6.8) | 61.90 |
| 1’ | 4.41 (d, *J* = 7.8 Hz, 1H) | 99.82 | 4.41-3.27 | 4.41-99.82 |  | 4.32 (d, *J*=7.5, lH*)* | 100.80 |
| 2’ | 3.27 (m, 1H) | 73.68 | 4.40-3.27 | 3.27-73.68 |  | 3.24(m, lH) | 74.70 |
| 3’ | 3.41 (m, 1H) | 76.95 - 3.41 |  | 3.41-76.95 |  | 3.24 (m, lH) | 77.90 |
| 4’ | 3.34 (dt, *J* = 23.1, 8.6 Hz, 1H) | 70.56 |  | 3.34-70.56 |  | 3.34 (t, *J*= 8.3, 1H) | 71.50 |
| 5’ | 3.29 (m, 1H) | 76.95 |  | 3.29-76.95 |  | 3.24 (m, 1H) | 77.80 |
| 6’ | 3.86 (dd, *J* = 12.3, 2.8 Hz, 1H) | 61.80 |  | 3.86-61.80 |  | 3.77 (dd, *J*=12.1, 6.8, 1H) | 62.70 |

**
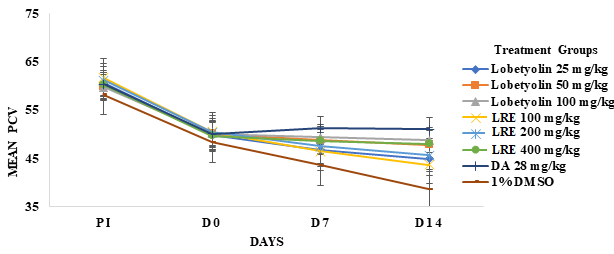
**

**Supporting Information 10: The effects of LRE and lobetyolin on body weight of *Trypanosoma congolence*-infected mice.**


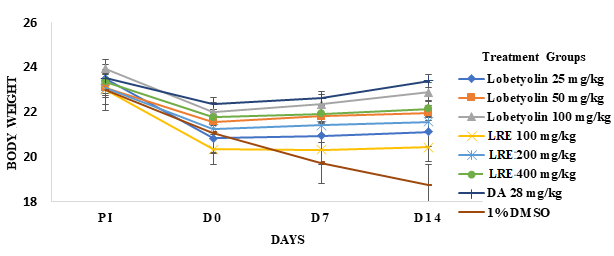


**Supporting Information 11: Effects of LRE and** **lobetyolin on body weight of *Trypanosoma congolense* infected mice.**

**
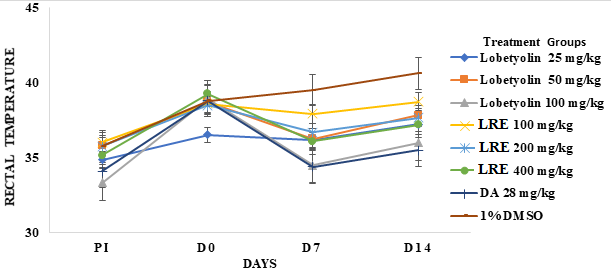
**

**Supporting Information 12: Effects of LRE and lobetyolin on rectal temperature of *Trypanosoma congolense* infected mice.**
